# Development and validation of a LASSO prediction model for cisplatin induced nephrotoxicity: a case-control study in China

**DOI:** 10.1186/s12882-024-03623-w

**Published:** 2024-06-11

**Authors:** Jingwei Zhang, Xuyang Luo, Yi Fan, Wei Zhou, Shijie Ma, Yuwei Kang, Wei Yang, Xiaoxia Geng, Heping Zhang, Fei Deng

**Affiliations:** 1 Department of Blood Transfusion, Chengdu Second People’s Hospital, Chengdu, China; 2 Department of Nephrology, Sichuan Provincial People’s Hospital, University of Electronic Science and Technology of China, Chengdu, 610072 China; 3 Department of Nephrology, Sichuan Provincial People’s Hospital Jinniu Hospital, Chengdu Jinniu District People’s Hospital, Chengdu, China; 4 Department of Nephrology, Affiliated Hospital of North Sichuan Medical College, Nanchong, China; 5 Department of Nephrology, Affiliated Hospital of Southwest Medical University, Luzhou, China; 6 Department of Elderly Infection, Sichuan Provincial People’s Hospital, University of Electronic Science and Technology of China, Chengdu, China; 7 Clinical Immunology Translational Medicine Key Laboratory of Sichuan Province, Sichuan Provincial People’s Hospital, University of Electronic Science and Technology of China, Chengdu, China

**Keywords:** Cisplatin, Nephrotoxicity, Prediction model, Lasso regression

## Abstract

**Background:**

Early identification of high-risk individuals with cisplatin-induced nephrotoxicity (CIN) is crucial for avoiding CIN and improving prognosis. In this study, we developed and validated a CIN prediction model based on general clinical data, laboratory indications, and genetic features of lung cancer patients before chemotherapy.

**Methods:**

We retrospectively included 696 lung cancer patients using platinum chemotherapy regimens from June 2019 to June 2021 as the traing set to construct a predictive model using Absolute shrinkage and selection operator (LASSO) regression, cross validation, and Akaike’s information criterion (AIC) to select important variables. We prospectively selected 283 independent lung cancer patients from July 2021 to December 2022 as the test set to evaluate the model’s performance.

**Results:**

The prediction model showed good discrimination and calibration, with AUCs of 0.9217 and 0.8288, sensitivity of 79.89% and 45.07%, specificity of 94.48% and 94.81%, in the training and test sets respectively. Clinical decision curve analysis suggested that the model has value for clinical use when the risk threshold ranges between 0.1 and 0.9. Precision-Recall (PR) curve shown in recall interval from 0.5 to 0.75: precision gradually declines with increasing Recall, up to 0.9.

**Conclusions:**

Predictive models based on laboratory and demographic variables can serve as a beneficial complementary tool for identifying high-risk populations with CIN.

## Introduction and background

Cisplatin and its analogues are widely used in chemotherapy regimens for cancer treatment, with approximately 10-20% of cancer patients receiving such treatment. However, the side effects of cisplatin can lead to reduced dosage or the selection of alternative therapies, ultimately affecting prognosis. The lack of effective treatment measures to alleviate side effects, such as gastrointestinal problems, hematologic toxicity, neurotoxicity, and ototoxicity, can decrease the quality of life and increase medical costs [1]. Cisplatin-induced nephrotoxicity (CIN) is a common side effect affecting 20-45% of patients, which is also the main limitation for its use [2–4]. Chemotherapy itself can cause renal tubular injury, interstitial nephritis, and thrombotic microvascular disease [5]. As cisplatin uptake and excretion are mainly mediated by proximal tubule transporters, its accumulation in renal proximal tubule cells can lead to cell injury [2]. Up to now, risk factors associated with CIN include advanced age, smoking, type of cancer, comorbidities, baseline blood biochemical levels before chemotherapy (such as creatinine, albumin, cystatin, etc.), exposure to nephrotoxic drugs (such as iodinated contrast agents, long-term use of non steroidal anti-inflammatory drugs (NSAIDs), and gemcitabine), electrolyte disorders (low serum magnesium levels), alcohol intake, and high-dose cisplatin (≥ 50 mg/m2) per dose, Frequency of administration, cumulative dose, and insufficient hydration during administration [6, 7]. By investigating related pathological mechanisms, such as reactive oxygen species and mitochondrial dysfunction, cell death pathways, inflammatory responses, autophagy, and other related signaling pathways, researchers have identified differences in the genetic characteristics of key genes in CIN [2, 8–10]. However, variations in clinical features, laboratory and genetic results, and the weight of risk factors have been observed across different studies, and there is a lack of sensitive and specific CIN prediction biomarkers for both genetic and non-genetic factors [11]. These differences may be attributed to genetic variability among research subjects, disease types and protocols, inconsistencies in laboratory results and research design and the standardization of data analysis [1, 12].

Predictive models have been widely used to diagnose, treat, and evaluate prognosis by integrating non-unique factors and comprehensively assessing their weight [13]. Such models may help identify individuals at risk of nephrotoxicity, guide optimal drug and dose selection, and inform prevention strategies. Given the objectivity of tumor genetic heterogeneity, it is necessary to construct a prediction model that combines prediction indicators based on more comprehensive clinical information and specific target gene information for unique types of tumors.

Genetic candidate genes and GWAS have identified several genetic risk factors for CIN [7, 11]. Okawa T [5] et al have developed a prediction model for CIN in elderly prostate cancer patients using a random forest algorithm that incorporated clinical and genomic characteristics extracted from saliva samples. It is believed that Genomic markers associated with nephrotoxicity are believed to be located in the regions between NAT1, NAT2, CNTN6, and CNTN4. Lung cancer remains the leading cause of cancer-related deaths worldwide, accounting for 30% of all cancer deaths in China [14, 15]. In terms of incidence, lung cancer is the most common cancer in China, with a mortality rate of 50% in Chinese males in 2020 [14]. Commonly recognized genetic variants associated with lung cancer and CIN include single nucleotide polymorphisms in genes such as ERCC1, ERCC2, and SLC22A2 [12]. In our study on mitochondrial pathway disorders, we observed a reduced risk of nephrotoxicity in carriers of the T allele of rs920829 in the TRAP1 gene compared to carriers of the C allele (OR 0.684, 95% CI 0.524–0.894, *p* = 0.003). Consequently, we plan to include SNP features of ERCC1, ERCC2, SLC22A2, and TRAP1 gene in future research.

The objective of this study is to utilize Lasso regression to identify suitable clinical and genetic features and construct and validate a CIN risk prediction model for lung cancer patients.

## Materials and methods

### Study subjects

**Fig. 1:**
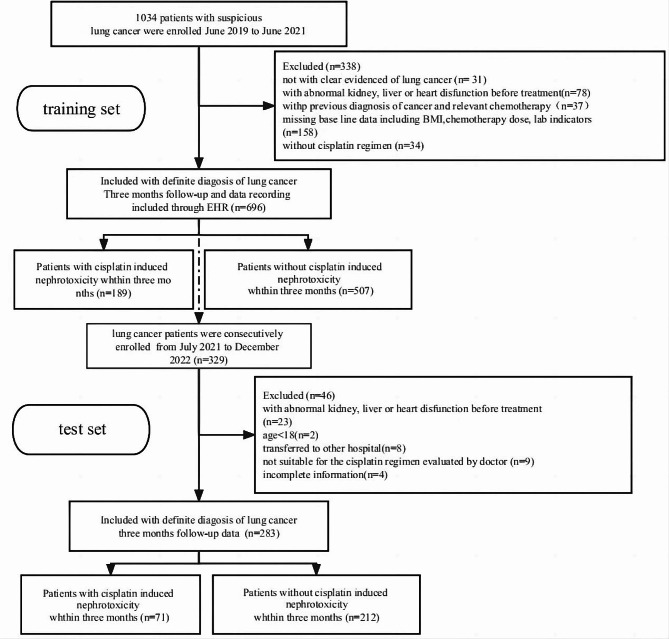
Flow diagram of the study population

A retrospective traing set was constructed to develop a predictive model for patients with clear lung cancer diagnosis and platinum chemotherapy regimen. The traing set included 696 patients who were hospitalized at Sichuan Provincial People’s Hospital between June 2019 and June 2021, of which 189 cases had CIN. A test set of 283 patients with lung cancer and platinum chemotherapy regimen was prospectively and continuously included from July 2021 to December 2022 in the same hospital. All patients underwent the same preliminary clinical evaluation and treatment observation. The research process was shown in Fig. 1.

Inclusion criteria were as follows: unrelated Han Chinese; having carboplatin-based chemotherapy; signed written informed consent; having demographic characteristics, physical examination, laboratory examinations, pathologically and histologically confirmed lung cancer; normal liver and kidney function before chemotherapy; and no obvious abnormalities in the preliminary clinical evaluation. Exclusion criteria included: <18 years old; liver or kidney dysfunction prior to initial chemotherapy [16]. This study conformed to the provisions of the Declaration of Helsinki (as revised in 2013) and it was authorized by the Ethics Committee of Sichuan Provincial People’s Hospital, University of Electronic Science and Technology of China Hospital. (Registration Number: AF-02/01.0). The chemotherapy regimens are listed in Table 1.

**Table 1:** Clinical characteristics and Indications for clinical laboratory tests of the study subjects

Group	Training set			Test set		
	Non-CIN (*n* = 507)	CIN (*n* = 189)	*P* value	Non-CIN (*n* = 212)	CIN (*n* = 71)	*P* value
**General data**						
Age (years) ^a^	45.65 ± 14.12	47.00 ± 12.45	0.219	45.51 ± 13.59	47.77 ± 11.95	0.213
BMI (kg/m^2^) ^b^	24.66(19.46–29.13)	24.09(20.41–27.51)	0.200	25.55(20.59–29.89)	23.86(20.32–28.36)	0.172
Gender (male/female) ^c^	244/263	99/90	0.932	123/89	45/26	0.489
Smoking (No/Yes) ^c^	178/329	74/115	0.331	79/133	25/46	0.778
Drinking (No/Yes) ^c^	135/372	56/133	0.446	52/160	22/49	0.280
Histology, n (%)						
Squamous cell carcinoma	289	109	0.863	111	44	0.232
Adenocarcinoma	139	48		54	19	
Undifferentiated carcinoma	42	19		32	6	
Others#	37	13		15	2	
Chemotherapy regimens						
cisplatin + Vinorelbine	72	22	0.294	15	7	0.619
cisplatin + Taxol/Docetaxel	113	49		58	19	
cisplatin + Gemcitabine	249	99		118	35	
cisplatin + etoposide	73	19		21	10	
Coronary heart disease	47/460	18/171	0.885	16/196	7/64	0.616
cerebrovascular diseases	50/457	22/167	0.487	20/192	9/62	0.498
hypertension	160/347	63/126	0.649	54/158	14/57	0.422
diabetes mellitus	155/352	56/133	0.853	61/151	23/48	0.552
**Laboratory examinations**						
Cisplatin-total ^b^	121.69(117.01-127.73)	128.19(109.60-142.15)	***< 0.001***	122.46(117.43–126.80)	120.13(96.51-136.79)	0.274
Cisplatin-average ^a^	62.65 ± 17.08	60.41 ± 19.71	0.170	63.20 ± 17.81	67.88 ± 24.31	0.138
RBC (×10^12^/L) ^a^	4.31 ± 0.93	4.28 ± 0.74	0.600	4.28 ± 0.90	4.22 ± 0.67	0.558
HCT (L/L) ^a^	0.38 ± 0.13	0.38 ± 0.14	0.781	0.39 ± 0.13	0.39 ± 0.13	0.735
PLT (×10^9^/L) ^a^	206.90 ± 96.72	204.73 ± 94.75	0.791	215.28 ± 99.77	211.45 ± 98.60	0.779
WBC (×10^9^/L) ^b^	6.74(6.06–7.58)	7.04(5.22–8.77)	0.325	6.57(5.79–7.25)	6.93(5.66–8.52)	0.112
Neu (×10^9^/L) ^b^	4.76(3.93–5.66)	4.96(3.21–6.56)	0.322	4.69(3.96–5.59)	4.90(3.47–6.16)	0.496
Mono (×10^9^/L) ^b^	4.30(3.50–5.18)	4.28(2.51–6.10)	0.616	7.78(6.83–8.63)	7.84(6.14–8.89)	0.102
Lym (×10^9^/L) ^b^	3.40(2.53–4.25)	3.18(1.91–4.91)	0.648	3.29(2.56–4.25)	3.16(1.85–4.75)	0.707
Neua (%) ^a^	64.69 ± 13.87	65.75 ± 11.21	0.303	65.17 ± 12.74	63.16 ± 9.80	0.169
Monoa (%) ^b^	7.71(6.88–8.64)	7.41(5.88–9.21)	0.055	7.78(6.83–8.63)	7.84(6.14–8.89)	0.755
Lyma (%) ^a^	22.23 ± 9.89	23.01 ± 9.32	0.350	23.01 ± 9.77	22.37 ± 7.85	0.578
TBIL (µmol/L) ^a^	10.00 ± 6.13	9.74 ± 6.97	0.651	9.56 ± 5.16	10.88 ± 7.84	0.188
DBIL (umol/L) ^b^	3.69(2.92–4.59)	4.44(2.92–6.23)	***< 0.001***	3.68(2.87–4.52)	4.69(2.92–5.95)	***< 0.001***
IBIL (umol/L) ^a^	4.68 ± 4.35	4.83 ± 4.87	0.706	5.02 ± 3.61	5.53 ± 3.52	0.303
ALT (IU/L) ^a^	17.17 ± 8.41	17.11 ± 5.09	0.912	17.70 ± 8.07	17.01 ± 4.51	0.371
AST (IU/L) ^b^	21.91(13.94–29.59)	22.94(15.78–29.13)	0.698	21.59(14.59–29.53)	23.02(15.82–27.46)	0.778
TP (g/L) ^a^	68.75 ± 8.22	70.56 ± 5.10	0.001	68.81 ± 8.42	70.66 ± 5.52	0.035
ALB (g/L) ^a^	40.75 ± 4.64	36.78 ± 5.35	***< 0.001***	40.98 ± 4.78	36.70 ± 5.12	***< 0.001***
GLB (g/L) ^a^	30.35 ± 9.33	30.79 ± 8.46	0.574	28.72 ± 9.57	32.39 ± 8.84	0.005
GLU (mmol/L) ^a^	5.57 ± 3.14	6.05 ± 3.12	0.073	5.89 ± 3.06	6.55 ± 3.27	0.123
UREA (mmol/L) ^a^	4.29 ± 1.00	4.34 ± 1.30	0.602	4.25 ± 0.98	4.44 ± 1.17	0.176
CREA (mmol/L) ^b^	60.90(38.86–85.13)	71.13(43.43–90.83)	0.018	65.01(48.04–87.74)	67.79(39.59–86.20)	0.927
CYS-C (mg/L) ^b^	0.71(0.64–0.77)	0.96(0.88–1.07)	***< 0.001***	0.73(0.64–0.79)	0.95(0.85–1.03)	***< 0.001***
URIC (umol/L) ^a^	301.38 ± 194.87	322.45 ± 198.37	0.207	325.20 ± 180.51	333.26 ± 200.64	0.752
GFR (ml/min) ^a^	91.63 ± 15.87	85.85 ± 14.14	***< 0.001***	91.06 ± 16.00	85.28 ± 15.57	0.008
TG (mmol/L) ^a^	1.14 ± 1.03	1.18 ± 1.20	0.664	1.31 ± 0.91	1.38 ± 0.92	0.568
CHOL (mmol/L) ^a^	4.95 ± 1.05	5.08 ± 1.01	0.155	5.03 ± 1.08	5.16 ± 1.02	0.376
HDL (mmol/L) ^a^	1.14 ± 0.53	1.13 ± 0.33	0.889	1.15 ± 0.54	1.16 ± 0.34	0.827
LDL (mmol/L) ^a^	2.38 ± 0.73	2.53 ± 0.71	0.018	2.39 ± 0.82	2.61 ± 0.66	0.038
ALP (IU/L) ^b^	78.95(65.99–93.98)	81.59(59.88-104.19)	0.458	77.94(64.64–91.33)	85.21(59.48-103.61)	0.225
GGT (IU/L) ^a^	35.20 ± 11.65	33.86 ± 11.81	0.180	34.72 ± 12.50	32.88 ± 9.42	0.194
LDH (U/L) ^a^	172.90 ± 44.70	187.46 ± 40.26	***< 0.001***	166.29 ± 41.28	188.83 ± 43.04	***< 0.001***
HBDH (U/L) ^a^	124.06 ± 33.51	123.24 ± 30.39	0.768	123.89 ± 31.06	125.22 ± 31.58	0.757
CRP (mg/L) ^a^	8.73 ± 6.16	10.37 ± 4.90	***< 0.001***	9.96 ± 5.50	10.92 ± 4.16	0.126
ESR (mg/L) ^a^	29.00 ± 15.84	30.00 ± 15.09	0.457	30.44 ± 15.52	30.37 ± 14.47	0.973
Na (mmol/L) ^a^	140.95 ± 14.67	138.44 ± 14.72	0.045	140.35 ± 15.67	142.51 ± 14.67	0.309
K (mmol/L) ^a^	4.20 ± 15.19	3.93 ± 15.08	0.834	12.35 ± 8.91	11.34 ± 8.70	0.406
Mg (mmol/L) ^a^	0.94 ± 0.23	0.85 ± 0.12	***< 0.001***	0.94 ± 0.24	0.86 ± 0.13	***< 0.001***
P (mmol/L) ^a^	1.27 ± 0.23	1.31 ± 0.23	0.016	1.26 ± 0.23	1.32 ± 0.23	0.037
Ca (mmol/L) ^a^	2.35 ± 0.22	2.35 ± 0.24	0.929	2.33 ± 0.21	2.35 ± 0.25	0.592

Complete response (CR), partial response (PR), stable disease (SD), and progressive disease (PD)

^a^ Data shown as mean ± standard deviation; ^b^data shown as median, interquartile range; ^c^ data shown as number of cases (frequency)

# Includes adenosquamocarcinoma, alveolus cell cancer and non-category NSCLC

BMI: Body Mass Index; RBC: Red Blood Cell; HCT: Hematocrit; PLT: Platelet; WBC: White Blood Cell; Neut: Neutrophil Count; Mono: Monocyte Count; Lym: Lymphocyte Count; Neua: Neutrophil Rate; Monoa: Monocyte Rate; Lyma: Lymphocyte Rate; TBIL: Total Bilirubin; DBIL: Direct Bilirubin; IBIL: Indirect Bilirubin; ALT: Aspartate Aminotransferase; AS;: Aminotransferase; TP: total protein; ALB: Albumin; GLB: Globulin; GLU: Glucose; CYS-C: Cystatin-c; GFR: Glomerular Filtration Rate; TG: Triglyceride; CHOL: Cholesterol; HDL: High Density Lipoprotein; LDL: Low Density Lipoprotein; ALP: Alkaline Phosphatase; GGT: γ-Glutamyl Transpeptadase; LDH: Lactate Dehydrogenase; HBDH: Hydroxybutyrate Dehydrogenase; CRP: C-reactive Protein; ESR: Erythrocyte Sedimentation Rate

### Definitions

Throughout each treatment cycle, toxicology information pertinent to the evaluation of cisplatin therapy (defined using the Common Terminology Criteria for Adverse Events version 5.0) was documented at least twice weekly [17]. This is the criteria how nephrotoxicity was rated: Grade 1, increased levels of creatinine above 0.3 mg/dL or 1.5–2.0 times higher than baseline levels; grade 2, 2–3 times higher than baseline levels; grade 3, more than 3 times higher than baseline levels or absolute levels above 4.0 mg/dL or requiring hospitalization; and grade 4, life-threatening consequences or requiring dialysis [17]. After 2 and 14 cycles, oncologic outcome reporting criteria were used to classify patient responses to treatment into 4 categories: complete response (CR), partial response (PR), stable disease (SD), and progressing illness (PD) [18].

### Data collection, preprocessing, and feature variable screening

The definitive diagnosis of CIN and basic medical history of subjects were exported from the HIS system by data collectors, and all relevant laboratory indications were exported in the LIS system. of complete blood count (SYSMEXXN-10, Sysmex, Japan), coagulation tests (SYSMEXCS-5100, Sysmex, Japan), and biochemical examination (Cobas c702, Roche, Germany)(Table 1). Candidate SNPs loci were typed using 48-Plex SNPscan® high-throughput SNP typing technology (18). Thirty samples were randomly selected for double-blind experiments to ensure the repeatability and stability of the genotyping results, and all the genotype calling success rates were greater than 99.0% [19]. For single variables measured multiple times, we retrieved patients’ admission records from the Hospital Information System (HIS) for those who underwent cisplatin chemotherapy regimens, and measurements, we retrieved patients’ admission records from the Hospital Information System (HIS) for those who underwent cisplatin chemotherapy regimens, and included their initial test records upon admission. The missing data of < 10% were filled with the median for continuous variables and plural for categorical variables, while missing data of > 10% were excluded. The medical records were used by data collectors to diagnose CIN, and any records without a definitive diagnosis were excluded after confirmation by a consulting clinician. Genetic polymorphism testing staff and clinical data collectors worked independently, and data analysts used all data jointly to build predictive models and perform performance validation. Absolute shrinkage and selection operator (LASSO) regression was used to initially screen candidate variables, with 1 standard deviation (1sd) penalty coefficient lambada (λ) selected.

### Identification of candidate predictors and construction of prediction models

The prediction model was constructed using multivariate logistic regression based on demographic variables and laboratory panel data [20]. . STATA software v15.0 was used to model candidate variables, with the goodness of fit evaluated using Akaike’s Information Criterion (AIC) [13, 21]. . The selection criteria were AIC minimization and candidate variable minimization without affecting predictive efficacy [21].

### Adjustment for model confounders and evaluation of predictive efficacy using training and test set data

Through 10-fold cross-validation, the model with the highest accuracy was selected. Covariance and interaction analyses were also performed on the candidate predictors. We used sensitivity, specificity, positive predictive value, negative predictive value, receiver operating characteristic (ROC) curves and C-index were used for model differentiation assessment, while calibration curve plots were used for consistency assessment [20]. 

### Statistical analysis

The clinical and laboratory data were analyzed using SPSS software (version 23.0). Quantitative data with normal distribution were analyzed using t-tests or ANOVA, while non-normal quantitative data were analyzed using Mann-Whitney or Kruskal-Wallis nonparametric tests. Count data were analyzed using the chi-square test or logistic regression [16]. Potential predictors were screened using Lasso regression in R version 3.6.1 software. Multi-factor analysis was performed using STATA version 14 software with logistic regression stepwise selection method, and the model was constructed based on the minimum AIC and the minimum number of predictors. Precision-Recall (PR) curve was plotted using the “ggplot2” package in R version 3.6.1 software. A nomogram was used to visualize the prediction model, and decision curves were used to analyze its clinical application value. The incidence of CIN in the China population was approximately 20% [22]. The bilateral significance level was set at 5%, with a test power of 80%. Taking into account a 10% loss to follow-up, the sample size for each group was estimated at approximately 100 cases [23].

## Results

### Basic information about the study population and clinical characteristics

In total, 979 patients were included in this study, with 696 patients (189 CIN vs. 507 controls) in the traing set and 283 patients (71 CIN vs. 212 controls) in the test set. There was no significant difference in the frequency of CIN between the two sets. Table 1 presents the clinical characteristics of the study subjects, while Table 2 displays the distributions of allele and genotype frequencies of all SNPs.

**Table 2:** The distributions of allele and genotype frequencies of all SNPs

Gene、	dbSNP			allele			genotype		
		allele	P^HWE^	CIN(n,%)	non-CIN(n,%)	P	CIN(n,%)	non-CIN(n,%)	P
				1/2	1/2		11/12/22	11/12/22	
ERCC1	rs11615	G > A	0.989	80/298	223/791	0.771	13/54/122	32/159/316	0.917
ERCC1	rs3212986	C > A	0.997	118/260	253/762	0.020	22/74/93	61/130/316	0.020
ERCC2	rs13181	T > G	0.091	37/341	96/918	0.838	8/21/160	17/62/428	0.802
ERCC2	rs1799793	C > T	0.640	33/345	82/932	0.743	5/23/161	13/56/438	0.232
ERCC2	rs238405	A > T	0.641	42/336	99/915	0.485	13/16/160	30/39/438	0.836
SLC22A2	rs316019	C > A	0.118	43/335	122/892	*0.780*	11/21/157	34/54/419	0.906
BACH2	rs920829	G > A	0.188	107/272	254/760	0.217	24/59/106	54/146/307	0.535
TRPA1	rs920829	C > T	0.883	86/292	291/723	0.030	16/54/119	86/119/302	0.017

### Model predictor screening

**Fig. 2:**
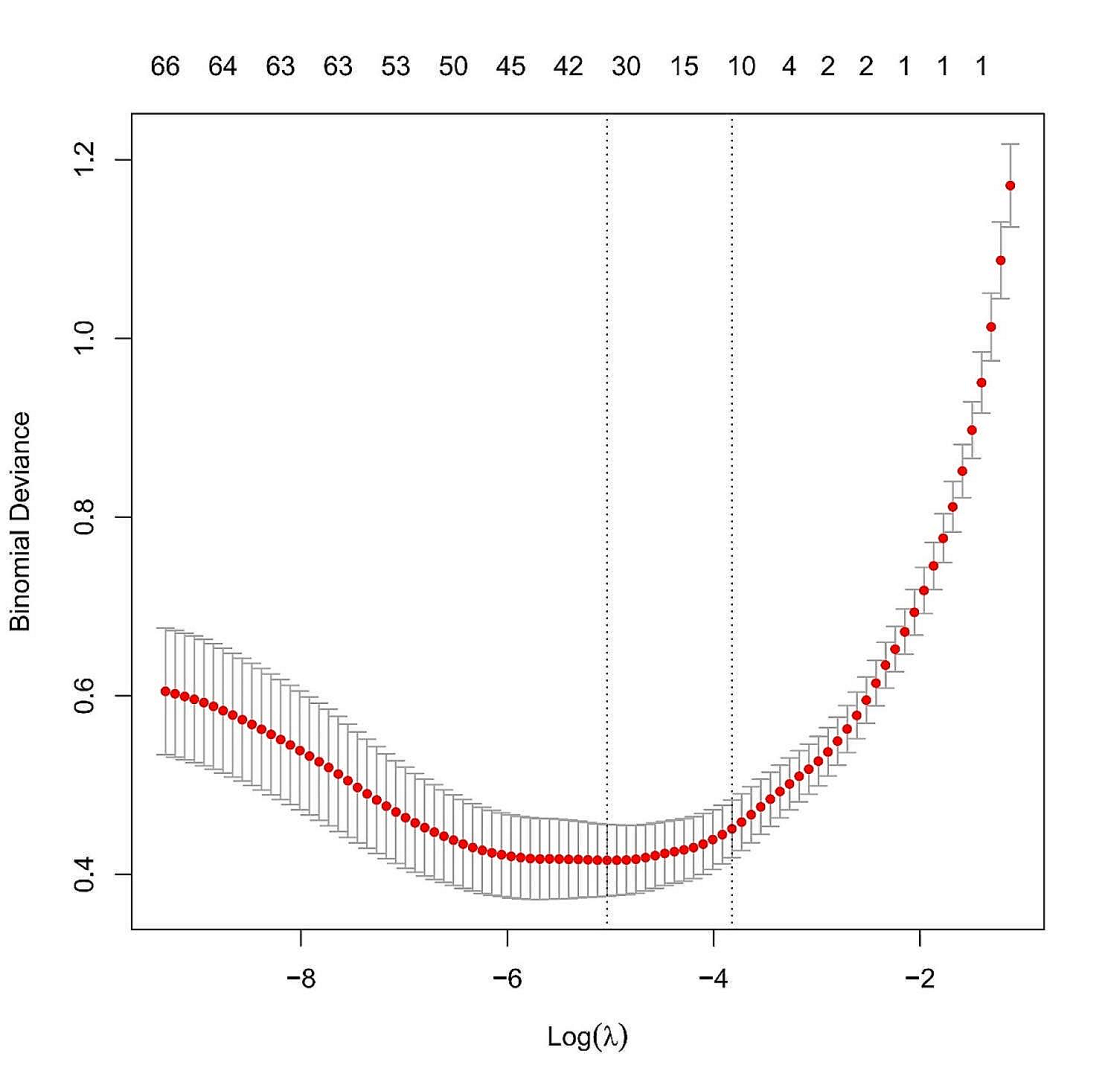
Determination of the optimal penalty factor λ = 0.006521281 (mininum) and λ = 0.02185674(1 Ssd) in the Lasso model using 10-fold cross-validation

**Fig. 3:**
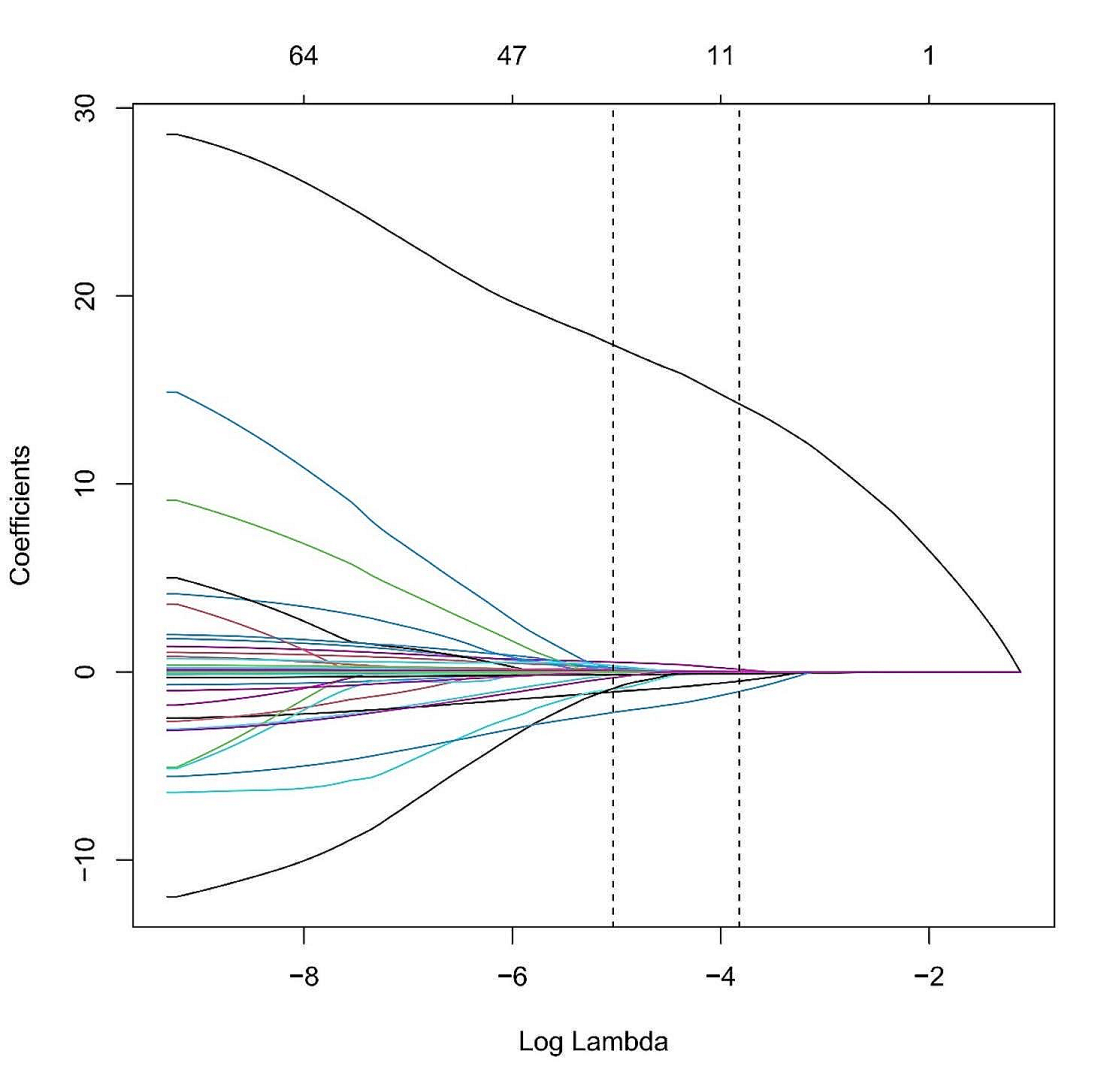
Distribution of Lasso coefficients for the 69 clinical characteristics. The left dashed vertical line shows the 36 non-zero coefficient variables for which λ was chosen as the minimum and 11 non-zero coefficient variables for which λ was chosen as the 1se

Lasso regression was utilized to screen variables in the traing set, revealing that the optimal subset of non-zero coefficient variables for inclusion in the model was 36 at the 1sd value of 10-fold cross-validation error λ = 0.02185674 and 11 at the minimum value of 10-fold cross-validation error λ = 0.006521281, as depicted in Figs. 2 and 3.

### Identification of candidate predictors and prediction model building

36 candidate predictors were modeled in various ways, and the screening *p* values, AIC, and BIC were presented in Table 3. Model 1 had the smallest AIC of 246.41, but it contained an excessive number of predictive factors. Model 2 incorporated 11 variables with an AIC of 274.35, model 4 incorporated 10 variables with an AIC of 285.48, and model 8 incorporated 9 variables with an AIC of 344.94. A comparison of model 2, model 4, and model 8 using the “lrtest test command” of STATA software revealed that although model 4 and model 8 incorporated fewer variables, their predictive efficacy was reduced (both *p* < 0.05). The inclusion of rs3212986 as a dummy variable in the predictive factors did not improve the predictive efficiency as the AIC and the number of predictive factors of the model increased. Therefore, model 2 was considered the best model with the characteristics of incorporated variables as shown in Table 3.

**Table 3:** Multiple models using multivariate logistic regression for comparison

Models	Construction method	Inclusion of variables	Screening	df	AIC	BIC
model1	Stepwise method	All variables	0.2	20	246.41	337.31
model2	Stepwise method(forward)	All variables	0.05	11	274.35	324.35
model3	Stepwise method (backward)	All variables	0.05	14	247.14	310.78
model4	Entry into law	rs3212986 cys p alb urea ca. dbil mg tp gfr	/	10	285.48	330.98
model5	Entry into law (dummy variable)	rs3212986 cys p alb urea ca. dbil mg tp gfr tp gfr	/	11	285.53	335.48
model6	Entry into law	cys alb urea ca. dbil mg tp gfr	/	9	344.64	385.55

AIC, Akaike’s information criterion; BIC, Baysian information criterion;

### Adjustment for model confounders and evaluation of predictive efficacy

In the adjustment for model confounders, interaction and collinearity were evaluated among the variables included in model 2 using the “corr test” command of STATA software. There was no interaction or collinearity between the predictors (data availabe if necessary). Logistic regression models were recreated in the test set data summary using the regression coefficients from the traing set model:

Odds(CIN)=1/(1+exp(-(6.62-2.191709*mg-0.1459131*alb-0.0252943*gfr-+0.0626123*tp-19.34694*cys+0.0132124*ldh+0.6151795*urea1+5.472858*p-0.686818*ca+0.3402039*dbil))).

**Fig. 4:**
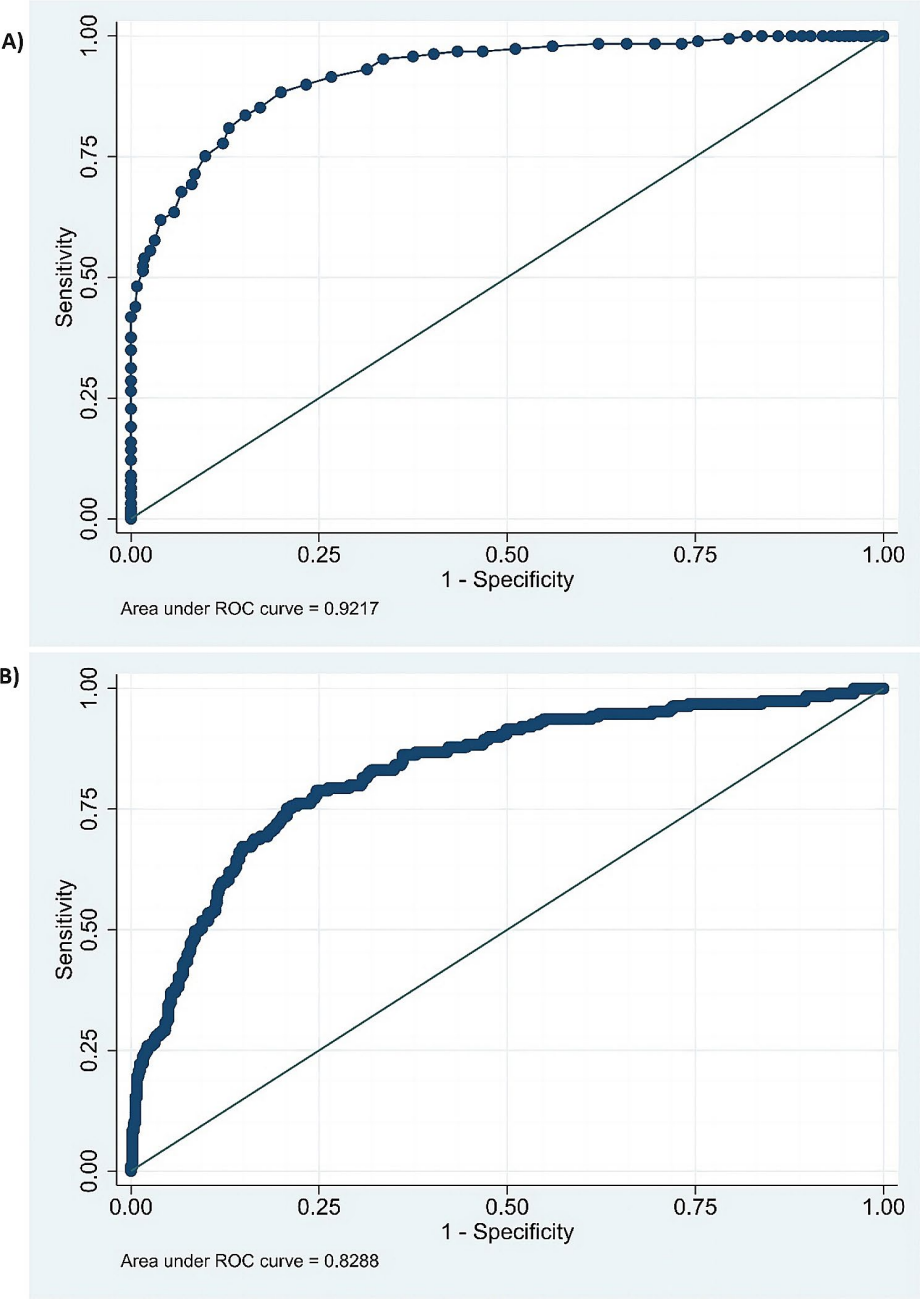
(**a**) ROC curve of the prediction model built from the training set data. The area under the curve is 0.9217, indicating good discrimination. ROC, receiver opertating characteristic. (**b**) ROC curves established by applying the CIN prediction model in the validation set. the area under the ROC curve is 0.8288, indicating good discrimination

**Fig. 5:**
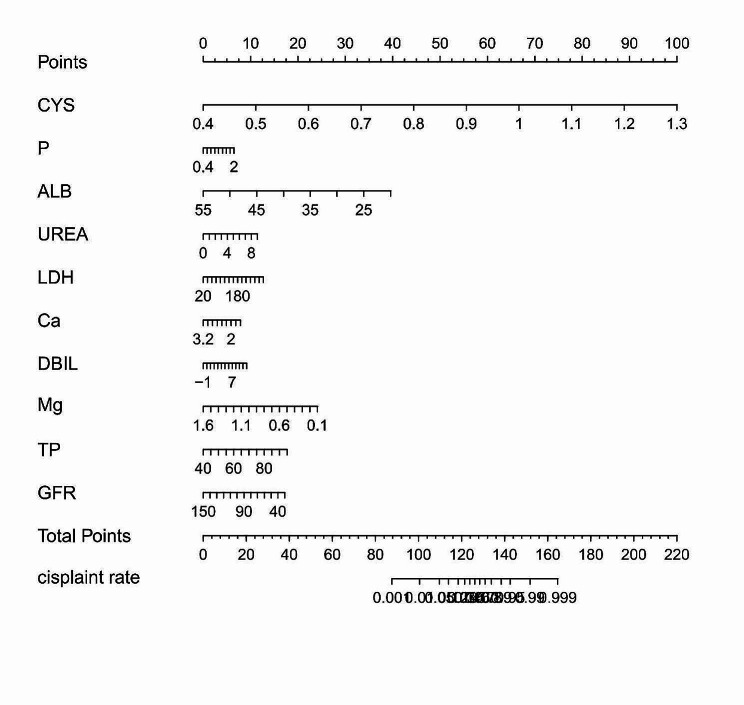
CIN prediction model presented as a column line graph plot

**Fig. 6:**
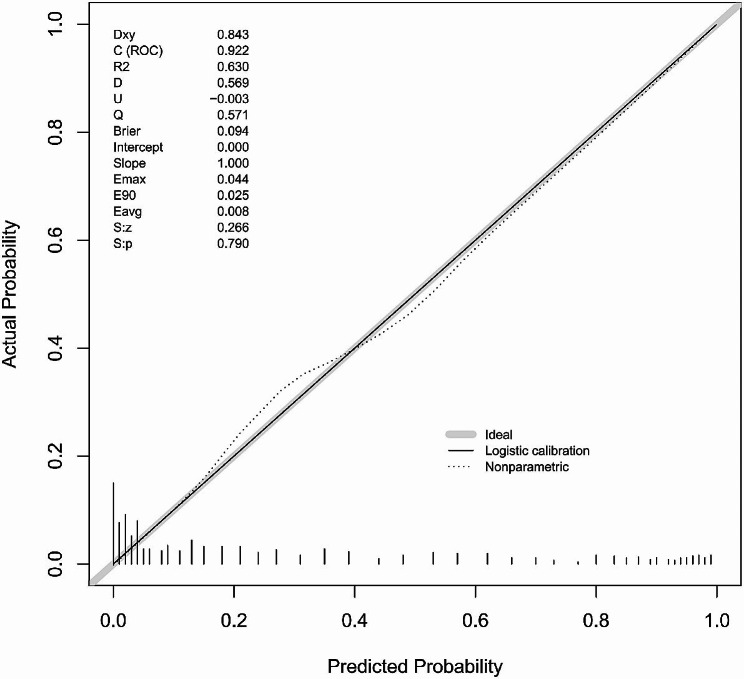
(**a**) Comparison of the agreement between the predicted risk of the CIN prediction model and the observed actual risk of the CIN in the training set. the gray straight line at 45° over the origin represents the ideal line; the gray dashed line represents the actual observed value and the black straight line represents the predicted value according to the logistic model, S:*p* = 0.790. CIN: cisplatin induced nephrotoxicity Dxy, Somer’s rank correlation between p and y: DXY = 2(C-0.5); C, ROC area; ROC, receiver opertating characteristic; R2 Nagalkerke-Cox-Snell-Magee R-saquard index; D, Discrimination index D; U, unreliability index; Q, the quality index; Brier, Brier score (average squared difference in p and y); Emax, maximum absolute difference in predicted and loess-calibrated probabilities; E90, the 0.9 quantile absolute difference in predicted and loess-calibrated probabilities; Eavg, the average quantile absolute difference in predicted and loess-calibrated probabilitie; S:Z, The Spiegelhalter Z-test for calibration accuracy; S:P, the two-tailed value of Spiegelhalter Z test

**Fig. 7:**
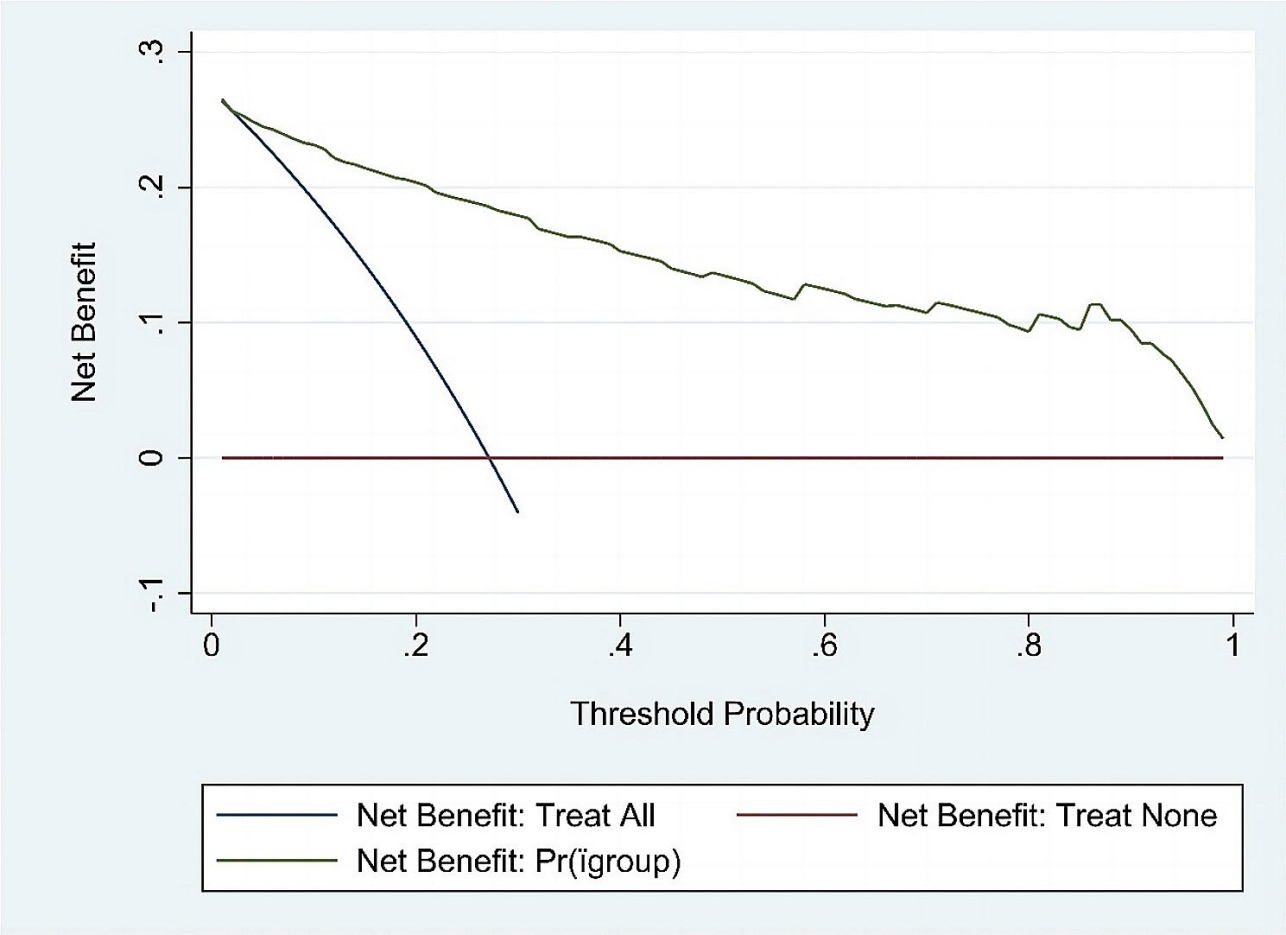
Clinical decision curves for the established CIN prediction model. The thin blue line is the net benefit of therapeutic intervention for all men; the thin green line is the net benefit of therapeutic intervention for the men on the basis of the statistical model; the thick black line is the net benefit of therapeutic intervention for no man. The threshold probalility of X-axis and Net benefit of Y-axis are displayed as a ratio. Pr, Threshold Probability

Table 4 presents the variables and characteristics that were ultimately included in Model 2. The predictive performance of the model is displayed in Table 5; Fig. 4, while the nomogram based on this prediction model is presented in Fig. 5. The agreement between the predicted and observed actual risk of CIN is compared in Fig. 6, and the clinical decision curve for the CIN prediction model is shown in Fig. 7. The model is deemed clinically valuable when the risk threshold ranges between 0.1 and 0.9.

**Table 4:** Variables and characteristics eventually included in the model

Characteristic variable	ß	OR	95% CI		*p*
			Lower limit	Upper limit	
cys	-19.34695	0.0012	0.0425	0.318	< 0.001
P	5.472858	0.0475	0.8153	0.9205	< 0.001
Alb	-0.1459131	0.8663	1.2428	2.1373	< 0.001
Urea	0.6151795	1.6298	1.0045	1.0180	0.001
Ldh	0.0132124	1.0112	0.0326	0.4677	0.002
Ca	-2.686818	0.0839	1.1819	1.6693	0.001
Dbil	0.3402039	0.1237	0.0254	0.5421	0.006
mg	0.1458457	0.0916	1.0107	1.0971	0.013
tp	0.0626123	0.0220	0.9554	0.9927	0.007
gfr	-0.0252943	0.0095	0.8153	0.9205	< 0.001

OR, odds ratio; CI, confidence interval

**Table 5:** Performance of prediction model in training and test set

	training set	test set
Sensitivity	79.89%	45.07%
Specificity	94.48%	94.81%
Positive predictive value	84.36%	74.42%
Negative predictive value	92.65%	83.75%
False positive rate	5.52%	5.19%
False negative rate	20.11%	54.93%
Correctly classified rate	90.52%	82.33%
Area under ROC curve	0.9217	0.8288

**Fig. 8:**
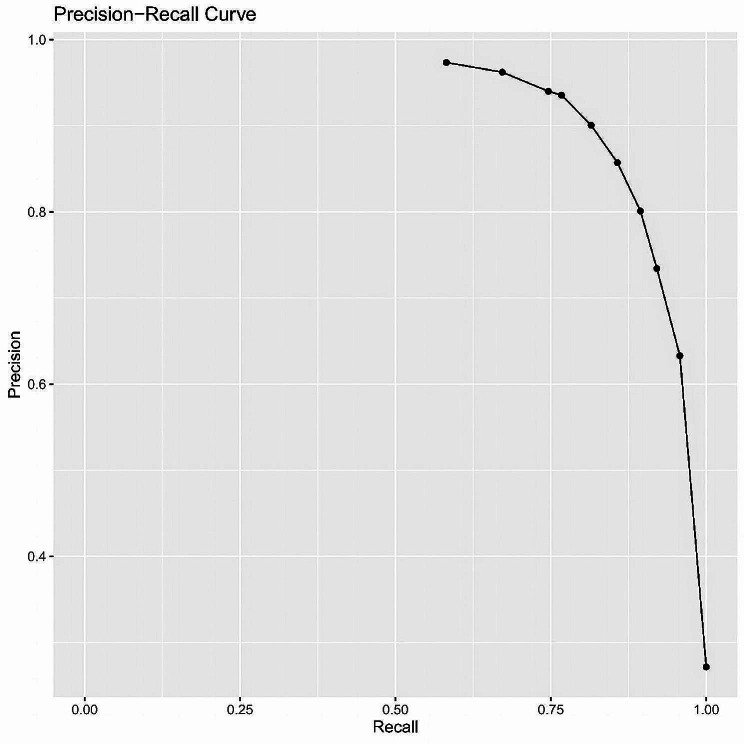
Precision-Recall (PR) curve of the predction model. The vertical axis in the figure represents accuracy, the horizontal axis represents recall, and the curves represent the corresponding accuracy and recall values at different cut-off points

Given the class imbalance, we used Precision-Recall (PR) curve for the assessment of the model’s predictive performance as shown in Fig. 8. In recall interval from 0.5 to 0.75: precision gradually declines with increasing Recall, remaining relatively high, up to 0.9. Within this range, the model maintains high accuracy in identifying positive samples and minimizing errors. In ecall interval from 0.75 to 0.90,precision drops more rapidly, from 0.9 to 0.60. To improve recall further and identify more positive samples, the model sacrifices more Precision, resulting in more false positives. In recall interval from 0.90 to 1.0,as recall approaches completeness, precision sharply decreases to about 0.10. In the pursuit of complete recall, the model’s accuracy significantly diminishes, introducing a large number of false positive predictions.

### Independent validation

The proposed model’s performance was evaluated using test set data, and its fit was consistent with that of the traing set data, as determined by the Hosmer-Lemeshow test (*p* = 0.4636). The overall predictive performance of the model is illustrated in Table 5; Fig. 4, and Fig. 6.

## Discussion

This study utilized machine learning algorithms to construct a CIN prediction model based on clinical, laboratory, and genetic variables. The construction process was conducted strictly to the statement of clinical prediction models as follows: developing the prediction model, validating the prediction model, and predictive effectiveness evaluation [24]. The model demonstrated good sensitivity and specificity, indicating that combining laboratory and clinical variables can effectively identify high-risk populations of CIN. While the model cannot be used as an independent diagnostic method, it can serve as a supplementary tool due to its common, objective, and easily obtainable predictive factors.

The predictive set factor included 69 feature variables, 8 of which were genetic. If the genetic variables were considered as dummy variables, the total number of variables would increase to nearly 80. we employed LASSO regression with a 1sd penalty coefficient to consolidate the laboratory variables. This method effectively reduced the number of predictors and eliminated unimportant variables. LASSO is a method of shrinkage estimation based on model reduction. By constructing different penalty functions, the regression coefficients of variables will decrease accordingly, and the regression coefficients of unimportant variables will eventually decrease to zero. Compared with the classical screening method, Lasso can effectively avoid the influence of factors such as different orders of magnitude, different units and possible collinearity between variables [25]. To screen candidate variables, we opted for Lasso regression over classic single factor regression, using a 1 standard deviation penalty coefficient lambda (λ) as the screening parameter to prevent the exclusion of relatively unimportant variables [7, 26, 27]. The LASSO algorithm was executed using the “glmmet” R package, while the logistic regression model was constructed using the “glm” R package [20]. Subsequently, we employed multifactor logistic stepwise regression to identify a concise and effective set of variables, which were then fitted into the formula based on their respective weights. This standardized approach to variable selection and weight conversion helps mitigate differences in the same indicator arising from different laboratory methods [13, 28].

In the traing set, the genetic variable rs3212986 of ERCC1 exhibited statistically significant differences in allele frequency and genotype characteristics between the CIN group and the control group. The proportion of A-allele carriers was higher in the CIN group (31.21%) than in the control group (24.92%). The proportions of AA, CA, and CC genotypes were 11.64%, 39.15%, and 49.20% in the CIN group, and 12.03%, 25.64%, and 62.32% in the control group, respectively. These findings suggest that carriers of the A allele of rs3212986 are more likely to develop CIN, which is consistent with previous studies [29]. Similarly, the allele frequency and genotype characteristics of rs920829 of TRPA1 were also statistically different between the CIN group and the control group. The proportion of T allele carriers was lower in the CIN group (22.75%) than in the control group (28.69%). The proportions of TT, CT, and CC genotypes were 8.46%, 28.57%, and 62.96% in the CIN group, and 16.96%, 23.47%, and 59.57% in the control group, respectively. These results suggest that T allele carriers of rs920829 are less likely to develop CIN. However, during the optimization of variables through multiple factor logistic regression, neither rs3212986 nor rs920829 were incorporated. It is possible that these variables lack independent predictive power or their independent predictive value is not significant enough [30].

Cystatin-C (Cys-C) was identified as the independent risk factor with the highest odds ratio (OR) value in the prediction model, surpassing other factors in predictive performance. The reasons for the increase of Cys-C and the high risk of CIN are analyzed as follows: 1) Cys-C is produced by all nucleated cells in the body. Cys-C in the blood is filtered by the glomerulus, and is degraded through reabsorption of the renal tubules, and is not secreted through the renal tubules. The progress makes it a more effective indicator of early glomerular filtration function than creatinine, urea nitrogen, and other indicators [31, 32]. Secondly, Cys-C is a member of the cysteine protease inhibitor family and an imbalance between cathepsin and protease inhibitors may lead to tumor invasion and metastasis, which can also promote an elevation of Cys-C [33, 34]. Other factors in the model, such as dbil and LDH, were not traditional renal function indicators or related to cisplatin metabolism pathway, but may reflect changes in physiological or pathological pathways during the occurrence and development of CIN (such as secretion and excretion, inflammatory response, oxidative stress damage, and electrolyte imbalance) during the occurrence and development of CIN [27]. Therefore, using appropriate weighted models for joint evaluation can can aid in the earlier identification of CIN risks.

The model showed high sensitivity and negative prediction value(NPV), which can help to recognize the high risk of CIN and remind clinical attention to the selection of chemotherapy regimen and the compatibility with drug dosage. The results also showed a satisfactory discrimination ability and a prediction curve that is close to the actual curve, which indicates that the model can provide prediction results that are highly consistent with the actual ones to identify cases with high risk of CIN. The model had a C-index = 0.922 for the traing set’s discriminant test, with the consistency test S: *P* = 0.790, Emax = 0.044, Eave = 0.007 and S: *p* = 0.790, suggesting both the model’s discriminant and consistency were good. To avoid overfitting of the model due to random and systematic errors, a validation model was constructed from aother prospective dependent set data. The fitting of the model constructed from the test set data is consistent with the fitting of the model constructed from the traing set data. Further clinical decision curve analysis of the model revealed that the model was of good value for clinical use when the high-risk threshold was between 0.1 and 0.9. Meanwhile, Recision-Recall curve shown in recall interval from 0.5 to 0.75: precision gradually declines with increasing Recall, up to 0.9.

The prediction model developed in this study has certain limitations. Firstly, it is a single-center study, and although the test set data was prospectively included, the test set data was obtained retrospectively from the electronic medical record system. Consequently, there were unavoidable factors such as missing data, resulting in a final traing set of 696 patients, which may limit the model’s scalability and necessitate further multicenter research and external validation. Secondly, the study did not incorporate the latest CIN-related biomarkers, such as malondialdehyde (MDA), NADPH oxidases (NOX), or heme oxygenase 1 (HO-1), which could potentially impact the results [2]. Future research should focus on gradually conducting validation studies across multiple centers to continuously refine and enhance the model and provide guidance for clinical practice.

## Conclusion

Predictive models based on laboratory and demographic variables can serve as a beneficial complementary tool for identifying high-risk populations with CIN.

## Data Availability

All data generated or analysed during this study are included in this published article [and its supplementary information files].
